# Correction: Lipoprotein Ratios: Correlation With Thyroid Profile and Glycated Hemoglobin (HbA1c) Among Diabetes Mellitus Patients

**DOI:** 10.7759/cureus.c192

**Published:** 2024-09-13

**Authors:** Ayan Banerjee, Jagriti LNU, Prabhat LNU, Akash Bansal

**Affiliations:** 1 Biochemistry, All India Institute of Medical Sciences, Patna, Patna, IND; 2 Biochemistry, All India Institute of Medical Sciences, Gorakhpur, Gorakhpur, IND

This article has been corrected to fix several typos causing confusion regarding the study design and dates. The title has been changed from 'Lipoprotein Ratios: Correlation With Glycated Hemoglobin (HbA1c) Among Thyroid Disorders Patients' to 'Lipoprotein Ratios: Correlation With Thyroid Profile and Glycated Hemoglobin (HbA1c) Among Diabetes Mellitus Patients'. Additionally, the Materials & Methods section has been updated as follows:

- In the first paragraph, the study dates have been corrected from June 2022 - July 2022 to June 2023 - July 2023
- In the third paragraph, the following sentence has been revised to indicate a previous history of drug treatment: "Individuals with a previous history of hyperthyroidism, hypothyroidism, serious infections and malignancy, or those taking any drugs known to cause disturbance of lipid metabolism, were also excluded from the study." changed to "Individuals with a previous history of drug treatment for hyperthyroidism, hypothyroidism, serious infections and malignancy, or those taking any drugs known to cause disturbance of lipid metabolism, were also excluded from the study."

